# bioETH-PRS: confidential polygenic risk scoring with smart contracts on an FHE-enabled blockchain

**DOI:** 10.1093/bib/bbag503

**Published:** 2026-09-15

**Authors:** Kimon Antonios Provatas, Christos Galanopoulos, Ilias Georgakopoulos-Soares

**Affiliations:** Division of Pharmacology and Toxicology, College of Pharmacy, The University of Texas at Austin, Dell Pediatric Research Institute, Austin, TX 78712, United States; Division of Pharmacology and Toxicology, College of Pharmacy, The University of Texas at Austin, Dell Pediatric Research Institute, Austin, TX 78712, United States; Division of Pharmacology and Toxicology, College of Pharmacy, The University of Texas at Austin, Dell Pediatric Research Institute, Austin, TX 78712, United States

**Keywords:** blockchain, genomic privacy, FHE, smart contracts, risk categories

## Abstract

Polygenic risk scores (PRSs) aggregate genetic effect estimates to predict disease susceptibility, yet calculating one through an external service can require exposing raw genotype data. Homomorphic encryption hides those data during the calculation but, in prior work, still places a designated evaluator in a position of trust. We present **bioETH-PRS**, a protocol that replaces the evaluator with publicly auditable smart contracts on a blockchain supporting Fully Homomorphic Ethereum Virtual Machine (fhEVM). Using integer-exact encrypted arithmetic, bioETH-PRS computes the PRS dot product entirely in the encrypted domain, so genotype dosages and, at the model provider’s discretion, the GWAS weights stay hidden from the parties performing the computation. A fixed-point encoding represents signed weights as nonnegative integers within a bound that rules out overflow, recovering the score to the precision of the published weights. A four-contract architecture separates data custody, model publication, computation, and output release, and supports both a classic path that stores encrypted inputs and an appreciably cheaper streaming path that discards them. A release oracle can return a randomized risk category instead of the raw score, limiting what a repeated querier learns. Prototype evaluation on real GWAS fixtures, including a run on a public testnet, shows cost growing linearly with variant count and suggests the approach may be practical where transaction fees are low. Trust is redistributed rather than removed: the system still depends on the contracts, the blockchain, and the fhEVM services. We evaluate additive models of moderate size, not genome-wide or clinical use.

## Introduction

Advances in genome sequencing have made polygenic risk scores (PRSs) a central tool in precision medicine [1, 2]. A PRS aggregates the dosage-weighted effects of genetic variants:


(1) \begin{eqnarray*}& \textrm{PRS} = \sum_{i=1}^{N} g_{i} \cdot \beta_{i},\end{eqnarray*}

where $g_{i} \in \{0,1,2\}$ is the dosage of the model-specified effect allele for variant *i* and $\beta _{i}$ is the corresponding GWAS effect weight. Published polygenic scores in the PGS Catalog range from small panels to genome-wide scores with large variant sets [3]. Using a conventional external service to calculate a PRS can require sending genotype data outside the hospital, which raises privacy concerns because genomic sequences can be identifying [4, 5].

**The double-privacy problem.** The workflow handles two kinds of sensitive information: (i) *patient genotypes* ($g_{i}$), identifying, heritable, and permanent; and (ii) *GWAS model weights* ($\beta _{i}$), derived from restricted research cohorts. More generally, model interfaces and outputs can leak sensitive information through model-inversion attacks [6]. In a conventional plaintext service, the evaluator must decrypt both genotypes and weights before the calculation can proceed.

**The FHE opportunity.** Fully Homomorphic Encryption (FHE) permits arbitrary arithmetic on encrypted data without decryption. Knight *et al*. [7] demonstrate this with HEPRS, an open-source system that computes a 110 000-single-nucleotide polymorphism (SNP) schizophrenia PRS under the CKKS scheme [8] with near-zero accuracy loss (*r> 0.999*, MSE $< 2.3 \times 10^{-6}$). HEPRS, however, still uses a central evaluator: it relies on a three-party trust model (client, modeler, evaluator), and a colluding evaluator and client can reveal GWAS model weights. Trust in the evaluator is an assumption, not a protocol guarantee. Furthermore, HEPRS requires 3–4 GB of RAM per individual even at 110 000 SNPs, and output is a raw score—there is no built-in mechanism to prevent score accumulation or model probing.

**Figure 1:**
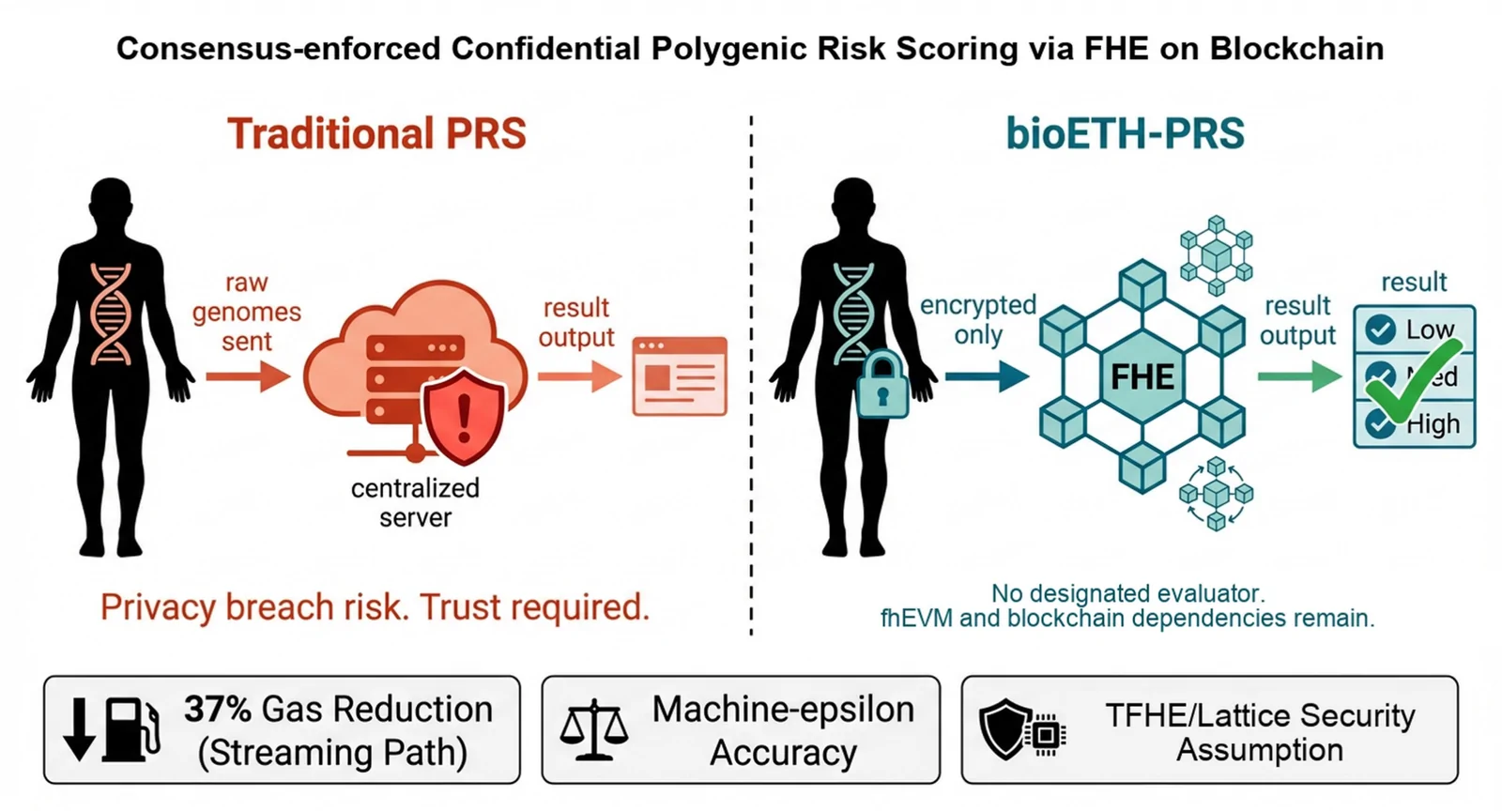
Graphical Abstract. bioETH-PRS utilizes smart contracts and fhEVM services to coordinate encrypted polygenic risk scoring calculations without a centralized evaluator, achieving exact reconstruction accuracy and up to 37.2% lower gas costs than traditional methods.

**Our contribution.** We propose **bioETH-PRS**, in which publicly auditable smart contracts coordinate the encrypted calculation and control how results are released. A designated evaluator is no longer required. However, the system still relies on the contracts, blockchain consensus, and the Fully Homomorphic Ethereum Virtual Machine (fhEVM) computation and decryption services (Fig. 1). The blockchain records the contract steps, but it does not by itself prove that the encrypted arithmetic was correct.

**Distinctions from HEPRS.** Although both systems compute the same encrypted PRS inner product, bioETH-PRS adds four design elements that are absent from HEPRS. First, TFHE/fhEVM exposes only unsigned integer arithmetic, requiring a dedicated fixed-point encoding and recovery scheme for signed GWAS weights; HEPRS instead uses CKKS with native approximate floating-point support. Second, bioETH-PRS separates data custody, model publication, computation, and output release into four independently auditable contracts. Third, it introduces two fhEVM-specific execution strategies, classic and streaming, that trade relay flexibility against gas and storage overhead under the handle/SSTORE model. Fourth, a model can require a randomized risk category and limit repeated queries instead of releasing the raw score.

Our main contributions are:

- Four independently auditable smart contracts that separately manage who may use a sample, model publication, encrypted calculation, and result release.
- A three-step conversion that represents signed decimal weights as nonnegative integers, checks that the calculation stays within the allowed integer range, and selects a suitable scale before the model is published.
- The Classic method (stored inputs) and Streaming method for processing the encrypted inputs; the Streaming method used about 37% less gas in the local simulation.
- A randomized risk category that can be required instead of releasing the raw score.
- Query limits and thresholds fixed by the model provider, evaluated with queries chosen after earlier results or in advance, several wallets and samples, and correlated SNP patterns.
- A public-weight 100-SNP calculation on Sepolia, public-weight calculations with 100–5000 variants in the local simulation, and one local private-weight 100-SNP calculation.

The intended use is research with additive PRS models containing up to 5000 variants. We did not evaluate genome-wide or clinical use, production cost, or private weights on Sepolia.

## Background

### Polygenic risk scores

A PRS is the weighted inner product of a patient’s effect-allele dosage vector $\mathbf{g} \in \{0,1,2\}^{N}$ and a GWAS effect weight vector $\boldsymbol{\beta } \in \mathbb{R}^{N}$ (Equation 1). Effect weights are typically estimated by large-scale GWAS over tens of thousands of individuals and are signed floating-point values whose range depends on the model. Standard pipelines such as PLINK 2 [9] and LDpred2 [10] compute PRSs in milliseconds on plaintext data; FHE approaches trade runtime for privacy.

PRS models span a wide range in SNP inclusion depending on construction methodology. Sparse or clinically oriented models may include hundreds to a few thousand variants, while genome-wide approaches incorporate tens of thousands to millions of SNPs. We evaluated bioETH-PRS with models containing up to 5000 variants. These results do not establish genome-wide or clinical use.

The computation in Equation 1 is an inner product between two vectors of moderate length. This is exactly the primitive that FHE systems are designed to support efficiently, making PRS computation a natural target for FHE-based privacy enhancement.

### Genotype preprocessing, QC, and model alignment

The pre-encryption workflow scores one individual against an already developed model. Minor-allele frequency and Hardy–Weinberg filtering are therefore cohort- and model-development quality-control operations performed upstream when effect weights are derived. The scoring procedure instead checks missingness, genome build, variant identity and order, allele orientation, and the dosage representation before any value is encrypted.

Only diploid hard-call dosages in $\{0,1,2\}$ are accepted. A non-integer or out-of-range dosage is rejected rather than clamped, because clamping silently changes the score. The model must state one of three rules for missing values: reject the sample, use dosage 0, or use a supplied cohort mean rounded half away from zero to a hard call in $\{0,1,2\}$. An unstated zero is avoided because it would be indistinguishable from a genuine homozygous-reference call. The declared genotype build must equal the model build; a mismatch is fatal because the build cannot be inferred from dosage values. Variant identifiers and order are checked element by element, not merely by vector length. Duplicate identifiers, multiallelic sites, and indels are rejected; this study evaluates biallelic SNP hard calls only.

Effect-allele alignment does not reveal encrypted weight magnitudes. Publicly visible model information supplies each variant identifier, genome build, effect allele, other allele, and column order, and alignment runs locally before encryption. For a biallelic, non-palindromic SNP, a dosage already counting the effect allele is retained; a dosage counting the other allele is transformed as $g_{\textrm{effect}}=2-g$. A compatible strand complement is applied before the same decision. Palindromic A/T or C/G pairs without explicit strand resolution are rejected even on a literal allele match, because the same label is consistent with opposite strand orientations. Any remaining incompatible pair is rejected.

The preparation record reports how many variants were matched, missing, imputed, rejected, or changed for allele orientation. The HEPRS data sets contain numeric matrices without variant identifiers, builds, or allele labels and are therefore treated as already aligned. Their leading constant column has weight 0 and dosage 1, so an *N*-variant data set contains *N+1* encoded positions. Examples with variant metadata covered the build, order, missingness, and strand rules.

### Fully homomorphic encryption

FHE schemes differ in the arithmetic they support and their performance. Two are directly relevant to this work.

**CKKS** [8] supports approximate arithmetic over real-valued data, is naturally suited to floating-point computations, and underpins HEPRS. Its available calculation depth depends on the selected parameters and whether bootstrapping is used, and approximation error accumulates during calculation. The Lattigo library [11] implements CKKS in Go with the parameter sets used by HEPRS. CKKS is computationally expensive: evaluating a 110 000-SNP PRS on a single CPU node requires *∼*4.9 s per individual and up to 130 GB of RAM for a cohort of 1000 [7].

**TFHE** [12] supports bootstrapped operations on encrypted values to arbitrary computation depth. Zama’s fhEVM implements TFHE as EVM precompile operations exposed to smart contracts, and the interface used here exposes exact unsigned-integer arithmetic. Exact integer arithmetic is a better fit for smart contracts, where bit-exact reproducibility is required for consensus. The cost is that signed floating-point weights must be converted to unsigned integer encodings, motivating our quantization design (Section Representing decimal weights as integers).

The security of TFHE is based on LWE/GLWE-family assumptions [12]; CKKS uses related lattice assumptions. These assumptions are generally studied as candidates for security against quantum attacks, but the present study does not evaluate long-term cryptographic parameter selection.

### Programmable blockchain and fhEVM

The fhEVM extends the Ethereum execution environment with TFHE precompile operations [13]. Every encrypted value is represented on-chain by a 32-byte opaque handle; actual ciphertexts are managed by an off-chain coprocessor. Smart contracts store only handles and trigger FHE operations through coprocessor calls.

An on-chain contract records the release permission for each result. If the fhEVM services work as specified, only the requester can decrypt a raw score authorized by the contract, whereas a randomized category is publicly decryptable. The blockchain records these decisions and calls, but it does not independently verify the encrypted calculation or prevent a failure of the fhEVM calculation and decryption services.

A per-transaction Homomorphic Computation Unit (HCU) budget limits FHE operations. In the local simulation, the largest successful calculation chunk contained 21 SNPs for both model types (Section Empirical evaluation); the Sepolia limit remains unmeasured and is not inferred from that result. The HCU constraint necessitates chunked computation strategies for large input vectors.

## System design

### Architecture overview

**Figure 2:**
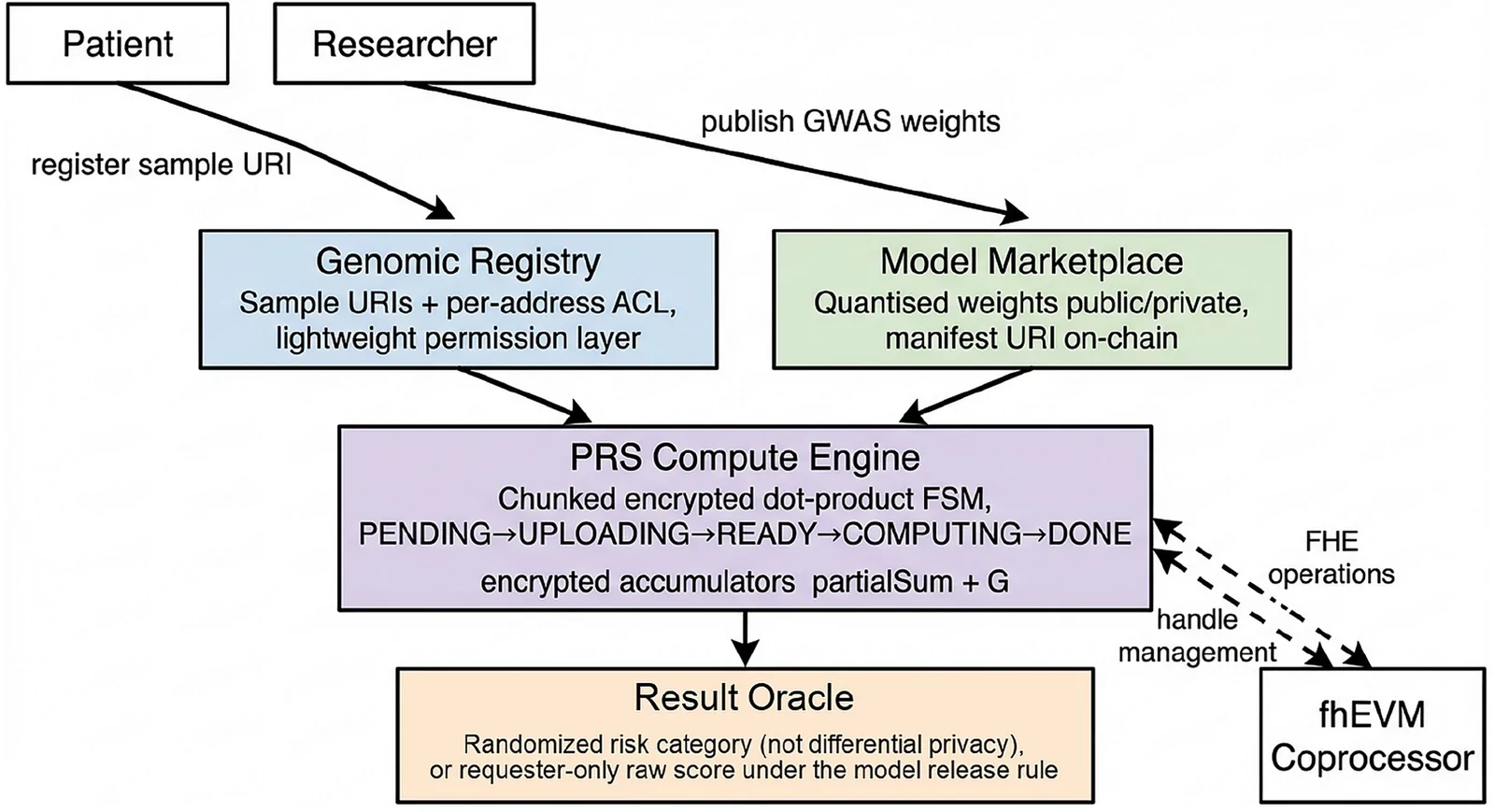
System architecture. Four contracts record who may use a sample, store the model and its result settings, calculate the encrypted score, and return either the score or a randomized risk category. Public weights and model details are visible by design; genotype dosages and private weights remain encrypted during the calculation. Raw scores are authorized only for the requester; randomized categories are publicly decryptable.

bioETH-PRS is structured as four independently deployable smart contracts forming a linear pipeline from sample data custody to risk output (Fig. 2). The four-layer separation reflects distinct roles and upgrade lifecycles: GenomicRegistry records who may use a sample, ModelMarketplace stores the GWAS model and its result settings, PRSComputeEngine calculates the encrypted score, and ResultOracle can release a randomized risk category. Each contract is independently auditable and can be replaced without affecting the others.

**Genomic registry.** URI-based registry of sample datasets with per-address access control lists (ACLs). It stores references (IPFS or Arweave URIs) to off-chain encrypted genotype data. A patient registers samples and explicitly grants compute access to specific callers. This contract performs no FHE operations; it is a lightweight permission layer.

**Model marketplace.** The model contract stores GWAS weights in chunks. Public weights are visible integers; private weights are encrypted handles. Public weights are encrypted before multiplication in the present implementation. A manifest URI records the quantization metadata on-chain: scale factor, weight zero-point, score offset, and a cryptographic fingerprint of the input-preparation record. Weights, quantization parameters, and the release policy cannot be changed after model finalization, but the owner can update or disable the query-rate limits. Public-weight calculations with 100–5000 variants and one private-weight 100-SNP calculation were evaluated locally. Only the public-weight 100-SNP calculation was performed on Sepolia.

**PRS compute engine.** The core computation contract. It implements a finite state machine (PENDING  *→*  UPLOADING  *→*  READY  *→*  COMPUTING  *→*  DONE) that orchestrates chunked dot-product accumulation of patient SNPs against model weights. The engine tracks two encrypted running accumulators: the weighted partial sum and the genotype sum required for the correction described in Section Representing decimal weights as integers. It finalizes either one raw score or one randomized category. A raw score is authorized only for the requester. A legacy readPartial function can separately authorize the requester to inspect the intermediate accumulator when the model does not require the oracle; oracle-required models block this path. We did not use readPartial in the reported evaluation. Section Execution protocols describes the two calculation methods.

**Result oracle.** The result contract adds a random value to the encrypted score before assigning Low, Medium, or High risk. The contract obtains the bounded encrypted random value from FHE.randEuint64. Its unpredictability is an assumption about the fhEVM services rather than a property independently verified by blockchain consensus. The model provider can require this category result instead of allowing the requester to decrypt the raw score.

### Comparison with HEPRS

Table 1 compares bioETH-PRS and HEPRS [7] by how they handle encrypted data, model size, runtime, memory, deployment needs, and the result returned. Both compute Equation 1. HEPRS reports encrypted CKKS results for a much larger model with a designated evaluator. bioETH-PRS reports a smaller calculation coordinated by public smart contracts. These contracts reduce reliance on one evaluator, but the system still depends on the blockchain and fhEVM services.

**Table 1:** Comparison with HEPRS. HEPRS values come from the published study; bioETH-PRS values state whether they were observed on Sepolia, observed in the local simulation, or not measured.

Dimension	HEPRS [7]	bioETH-PRS
How encrypted data are handled	encrypted client–modeler–evaluator system	encrypted inputs; contracts determine who receives the result
Designated evaluator	yes; evaluator executes CKKS	no designated evaluator; contracts coordinate the calculation
Remaining dependencies	three-party non-collusion and HE software/hardware	contracts, blockchain, fhEVM computation, and decryption services
Arithmetic scheme	CKKS approximate signed-real arithmetic	TFHE unsigned integers after fixed-point encoding
Encrypted variants evaluated	110 000 in the published study	public: 100 on Sepolia and 100–5000 locally; private: 100 locally
Runtime	*∼* 4.9 s/person at 110 000	269.320 s to result plus 8.081 s to decrypt at 100 on Sepolia; local times are not comparable
Memory	3–4 GB/person; up to 130 GB for 1000	not measured
Deployment needs	evaluator compute host and client/modeler setup	fhEVM blockchain, computation service, relayer, and decryption service
Result returned	raw score delivered to client; external controls optional	raw score or randomized risk category, as set by the model provider
Public information	model information managed off chain	model identifiers, alleles, order, result settings, and transactions are public; weights may be encrypted

## Representing decimal weights as integers

### The representation problem

TFHE arithmetic on the fhEVM operates on unsigned 64-bit integers. GWAS weights $\beta _{i}$ are signed floating-point values; a naive fixed-point encoding that multiplies by a scale factor *s* and rounds produces quantized weights $q_{i} = \textrm{round}(s \cdot \beta _{i}) \in \mathbb{Z}$, which may be negative. Negative integers cannot be stored in an unsigned 64-bit integer and would silently wrap under modular overflow, corrupting results without any observable error. A correct encoding must guarantee that all intermediate encrypted values remain in the nonnegative range $[0, 2^{64} - 1]$ for any genomically valid input $g_{i} \in \{0, 1, 2\}$.

### Three-step unsigned encoding

**Figure 3:**
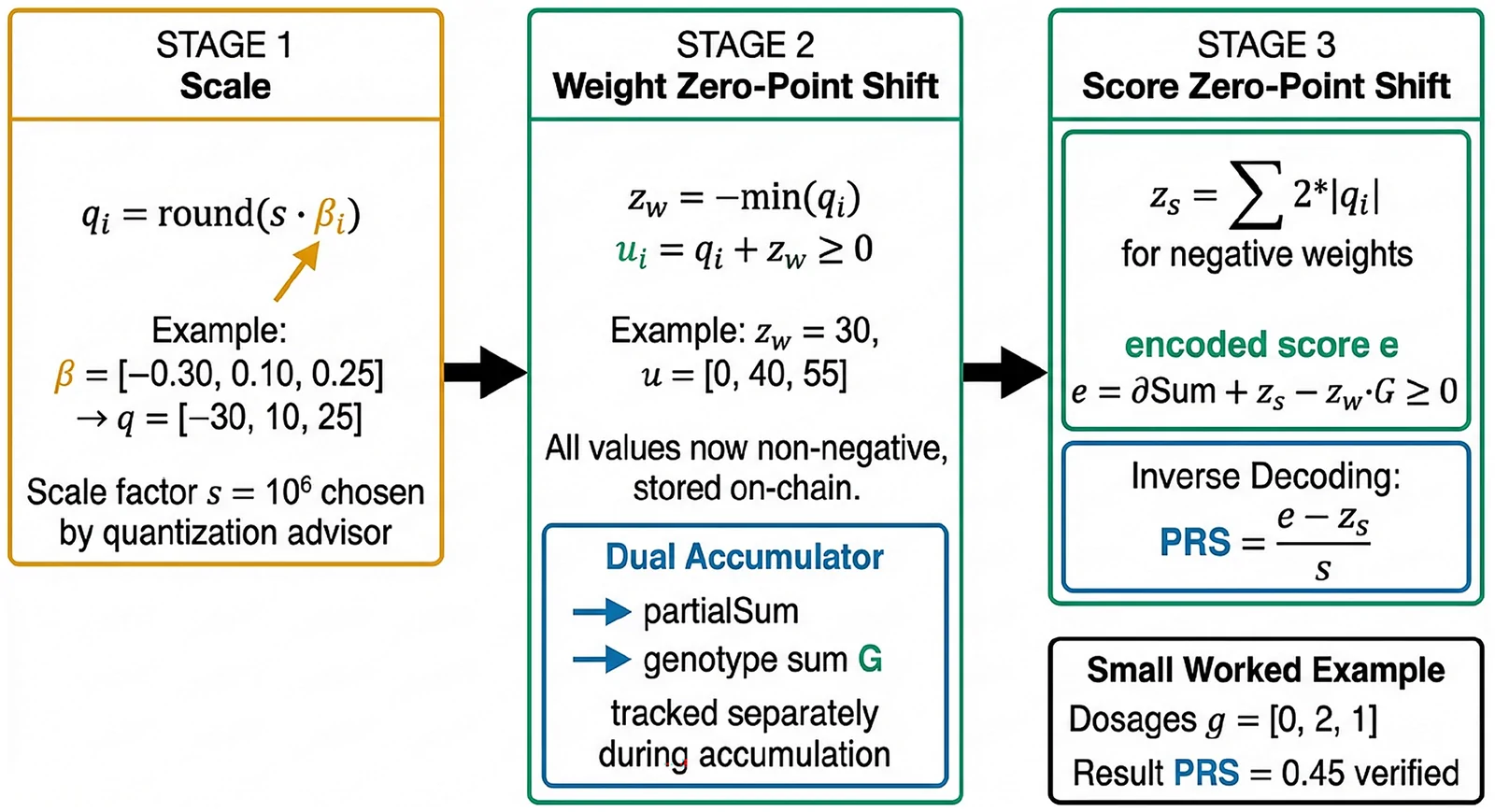
Original three-stage overview of the fixed-point conversion. The worked example is converted back to the additive PRS. Equations 3–6 give the exact half-away-from-zero rule, unsigned clamp, and intermediate-value order used in the implementation.

We convert the signed decimal weights to nonnegative integers in three steps (Fig. 3). The integer shifts are reversible. Conversion of the original decimal weights is exact only when the selected scale represents those weights without rounding, as it does for the data sets used here.

**Step 1: Scale.**


(2) \begin{eqnarray*}& q_{i} = \operatorname{round}_{\textrm{away}}(s \cdot \beta_{i}), \quad q_{i} \in \mathbb{Z},\end{eqnarray*}

where *s* is a model-specific scale factor selected by the quantization procedure (Section Quantization advisor) and exact half ties are rounded away from zero. The study data sets require no tie breaking: all weights have at most six decimal places and the selected scales are integer multiples of $10^{6}$, so their quantization is exact.

**Step 2: Weight zero-point shift.** Let


(3) \begin{eqnarray*}& z_{w} = \max(0,-\min_{i} q_{i}), \quad u_{i} = q_{i} + z_{w} \;\geq\; 0 \;\;\forall i.\end{eqnarray*}

The clamp is required by the unsigned on-chain representation when all weights are positive; algebraically, $z_{w}=0$ already preserves $u_{i}\geq 0$. The shifted weights $u_{i} \geq 0$ are stored on-chain. The raw accumulation $\sum _{i} g_{i} u_{i}$ overestimates the true PRS by the constant $z_{w} \cdot \sum _{i} g_{i}$, requiring correction. The engine tracks the genotype sum $G = \sum _{i} g_{i}$ as a second encrypted accumulator to enable this correction.

**Step 3: Score zero-point shift.** Even after the weight correction, the corrected sum $P = \sum _{i} g_{i} u_{i} - z_{w} G$ can be negative for patients with many risk-decreasing alleles. We define


(4) \begin{eqnarray*} z_{s} &= -\sum_{i:\beta_{i}<0} 2 q_{i} \;=\; \sum_{i:\beta_{i}<0} 2|q_{i}|, \end{eqnarray*}

(5) \begin{eqnarray*} e &= P + z_{s} \;\geq\; 0. \end{eqnarray*}

*e* is the *encoded score* stored encrypted on-chain. The on-chain computation is structured to avoid any intermediate negative value:


(6) \begin{eqnarray*}& e = \bigl(\textrm{partialSum} + z_{s}\bigr) - \bigl(z_{w} \cdot G\bigr),\end{eqnarray*}

where $z_{s}$ is added before subtracting $z_{w} G$, ensuring the intermediate value $(\textrm{partialSum} + z_{s})$ is always nonnegative.

**Decoding.** After an authorized requester decrypts a raw score:


(7) \begin{eqnarray*}& \textrm{PRS} = \frac{e - z_{s}}{s}.\end{eqnarray*}

### Worked example

Consider three genetic variants with scale *s=100*. Their weights and effect-allele dosages are


\begin{align*} & \boldsymbol{\beta}=[-0.30,0.10,0.25],\qquad \mathbf{g}=[0,2,1]. \end{align*}

The plaintext target is


\begin{align*} & \textrm{PRS}=0(-0.30)+2(0.10)+1(0.25)=0.45. \end{align*}

1. Quantize: $\mathbf{q} = [-30, 10, 25]$.
2. Weight shift: $z_{w} = 30$, $\mathbf{u} = [0, 40, 55]$.
3. Accumulate: partialSum $= 0{\cdot }0 + 2{\cdot }40 + 1{\cdot }55 = 135$; *G = 0 + 2 + 1 = 3*.
4. Correction: $P = 135 - 30{\cdot }3 = 45$.
5. Score shift: $z_{s} = 2{\cdot }30 = 60$; *e = 45 + 60 = 105*.
6. Decode: PRS *= (105 - 60) / 100 = 0.45*.

At the system level, the data preparer first checks variant identifiers, genome build, dosage validity, missing values, and effect-allele orientation. Only then are dosage values and, for private models, weight magnitudes encrypted. The requester selects an authorized sample and model, submits the encrypted groups for calculation, and receives either the model-configured raw score or randomized category. The contracts record permissions and the result recipient, but they do not establish that the encrypted values came from the stated biological sample.

### Overflow safety

Proposition 1. Let *N* be an arbitrary SNP count, let *s* be the fixed-point scale factor, and let *M* satisfy $|q_{i}| \leq sM$ for every quantized weight $q_{i}$. Then a sufficient worst-case condition for unsigned 64-bit safety of the full encoding calculation is

\begin{align*} & 4 s M N \leq 2^{64} - 1. \end{align*}

Equivalently,

\begin{align*} & N \leq \left\lfloor \frac{2^{64} - 1}{4 s M} \right\rfloor. \end{align*}

Under this condition, both the final encoded score *e* and the intermediate quantity $\textrm{withOffset} = \textrm{partialSum} + z_{s}$ in Equation 6 fit in uint64.

Proof. Partition the quantized weights into positive, negative, and zero sets with cardinalities $N_{+}$, $N_{-}$, and $N_{0}$, so that $N_{+}+N_{-}+N_{0}=N$. Because $g_{i} \in \{0,1,2\}$ and $z_{w}=\max (0,-\min _{i} q_{i}) \leq sM$, the shifted weight $u_{i} = q_{i} + z_{w}$ obeys

\begin{align*} & u_{i} \leq \begin{cases} 2 s M, & q_{i}> 0,\\ s M, & q_{i} \leq 0. \end{cases} \end{align*}

Hence the encrypted accumulation before correction satisfies

\begin{align*} & \textrm{partialSum} = \sum_{i} g_{i} u_{i} \leq 4 s M N_{+} + 2 s M (N_{-}+N_{0}). \end{align*}

The score zero-point satisfies

\begin{align*} & z_{s} = -\sum_{i:q_{i}<0} 2 q_{i} \leq 2 s M N_{-}. \end{align*}

Therefore the intermediate value in Equation 6 is at most

\begin{align*} \textrm{withOffset} &= \textrm{partialSum} + z_{s} \\ &\leq 4sMN_{+}+4sMN_{-}+2sMN_{0} \\ &\leq 4sMN. \end{align*}

This is the worst-case upper bound for the intermediate sum.

For the final encoded score, observe that the weight-shift correction exactly cancels the zero-point contribution:

\begin{align*} & P = \textrm{partialSum} - z_{w} G = \sum_{i} g_{i} q_{i}. \end{align*}

Thus

\begin{align*} & -2 s M N_{-} \leq P \leq 2 s M N_{+}, \end{align*}

and after adding $z_{s}$ we obtain

\begin{align*} & 0 \leq e = P + z_{s} \leq 2 s M (N_{+}+N_{-}) \leq 2 s M N. \end{align*}

So the encoded score range itself fits in uint64 under the weaker bound $2 s M N \leq 2^{64}-1$, but the actual implementation must also accommodate the larger intermediate $\textrm{withOffset}$. A sufficient safety condition for all intermediate and final values is

\begin{align*} & 4 s M N \leq 2^{64}-1. \end{align*}

This condition was satisfied by every model evaluated in this study. It is an arithmetic safety check, not evidence that the system can process models larger than the evaluated maximum of 5000 variants.

### Quantization advisor

Before publishing any model, an automated quantization advisor evaluates the candidate scales


\begin{align*} & \begin{aligned} s \in \{&10^{2},3{\times}10^{2},10^{3},3{\times}10^{3},10^{4},3{\times}10^{4},\\ &10^{5},3{\times}10^{5},10^{6},3{\times}10^{6},10^{7}\} \end{aligned} \end{align*}

against the actual GWAS weight distribution. For each *s*, it computes the mean absolute error (MAE) between the quantized dot product and the floating-point PRS for all individuals in the study data set. It compares three tiers:

- **Low scale** ($s = 10^{2}$): MAE of 0.012–0.150 PRS units across the four fixture sizes.
- **Selected scale** ($s \approx 10^{6}$): zero error on these six-decimal-place data sets. A candidate recommendation for similarly represented models, not a universal production default.
- **Higher evaluated scale** ($s = 10^{7}$): no improvement over the selected scale on the study data; the limiting factor is source data precision.

The advisor should be run before model publication. In this fixed-euint64 implementation, changing the scale without changing the integer width does not change the contract operation count; the number of transactions needed to submit the SNPs remains the main scaling constraint.

## Execution protocols

**Figure 4:**
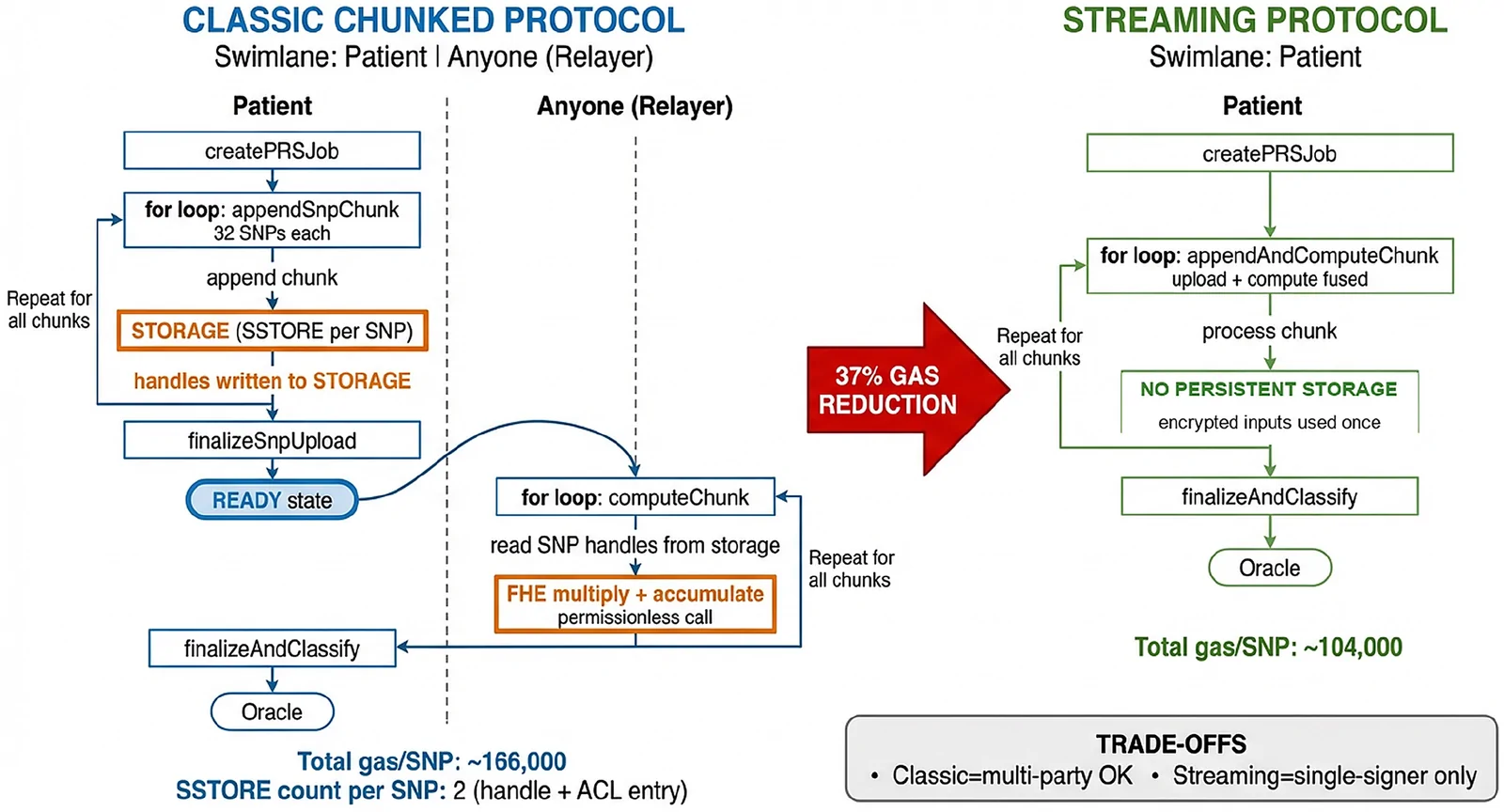
Calculation method comparison. The Classic method (stored inputs) uploads and stores SNPs before the score is calculated, so different parties can perform these steps. The Streaming method combines upload and calculation for one requester. Across the 100–5000 variants evaluated in the local simulation, streaming used 35.4%–37.2% less gas.

bioETH-PRS provides two computation strategies that differ in their gas cost, storage semantics, and support for multi-party participation, and in particular in whether encrypted SNPs are stored between transactions (Fig. 4). Both strategies produce bit-identical results.

### Classic method (stored inputs)

The classic protocol separates SNP ingestion from computation, enabling different parties to execute different phases at different times (Algorithm 1).



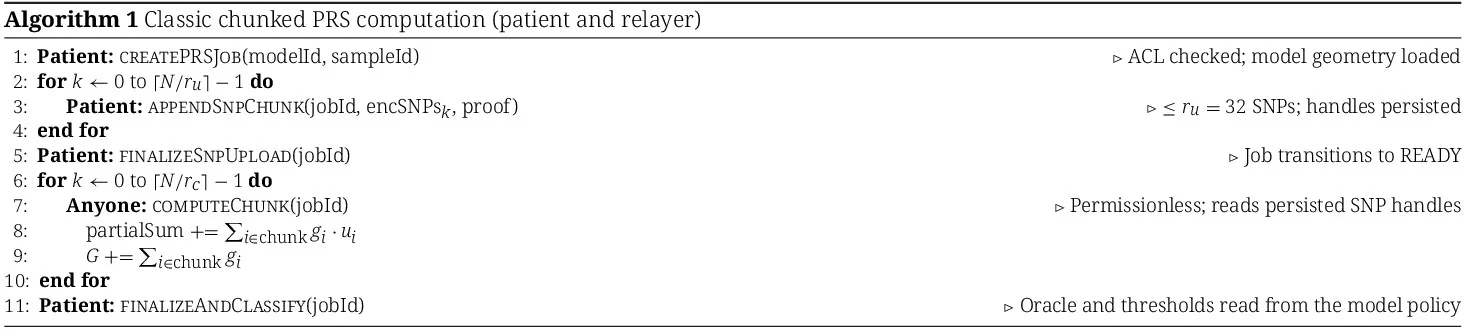



The upload chunk size $r_{u} \leq 32$ is constrained by the fhEVM input-proof budget (2048 bits/64 bits per encrypted integer). The compute chunk size $r_{c}$ is constrained by the HCU budget: each compute chunk of $r_{c}$ SNPs executes $3r_{c} + 2$ FHE operations (multiply, accumulate partial sum, accumulate genotype sum per SNP, plus two coprocessor calls for accumulator handles). The largest successful chunk in the local simulation contained 21 SNPs, and we used $r_{c} = 20$; the corresponding Sepolia limit was not measured.

SNP ciphertext handles are persisted in a flat per-job mapping across transactions, enabling upload and compute to be executed by different parties or at different times. The **compute phase is permissionless**: any third party, such as a relayer, may advance computation by calling the compute function. Under the encryption assumptions the relayer cannot read the SNP values, but it can delay requests, and its transactions reveal timing and addresses that are already visible on the blockchain. It therefore remains a dependency for availability and privacy.

### Streaming method

The streaming protocol fuses upload and computation into a single call per chunk (Algorithm 2). Each call immediately multiplies the incoming SNP handles against the model weights, accumulates the results, and discards the handles rather than storing them for later transactions. No per-SNP persistent storage writes are performed.



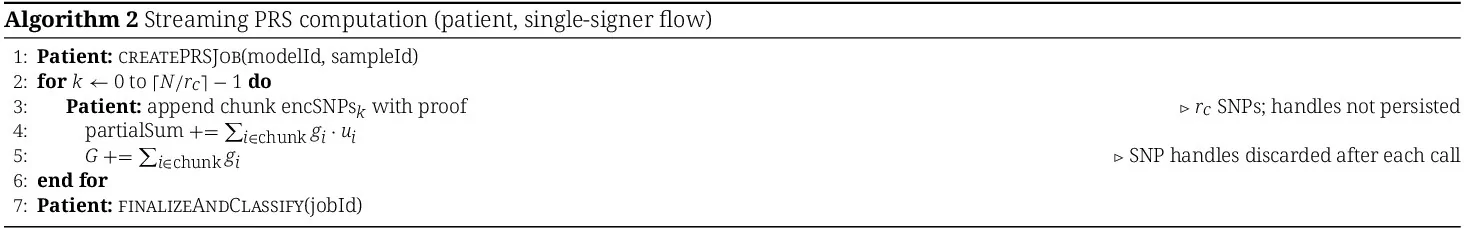



**Gas savings.** The classic path requires two persistent storage writes per SNP: a handle record in contract storage and an entry recording who may use it—each an Ethereum SSTORE at *∼*$25{\,}000$ gas. The streaming path avoids both, saving *≈*50 000 gas per SNP, which explains the observed 35.4%–37.2% reduction in local gas. The remaining *≈*95 000–104 000 gas/SNP in this implementation comes from input-proof verification, the FHE multiply, and the FHE accumulate—costs set by the fhEVM services rather than by contract design.

**Trade-off.** The streaming path is incompatible with multiparty upload/compute separation: upload and compute are coupled within the same transaction, restricting execution to a single signer. The classic path remains the appropriate choice for relay services and architectures where upload and compute are performed by different parties.

## Security assumptions and limits

### Threat model

**Figure 5:**
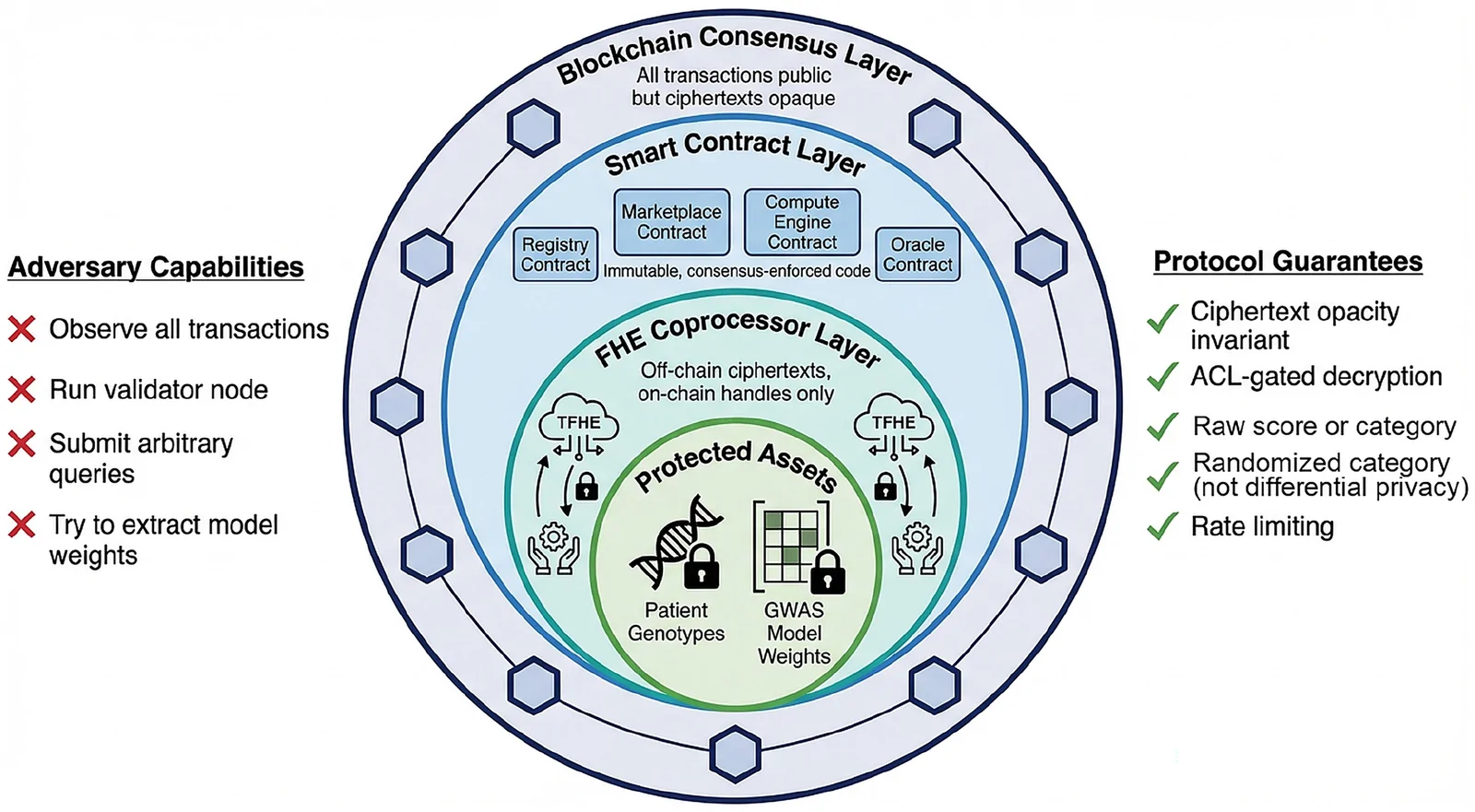
Security assumptions and remaining limits. The contracts control who may request a calculation, how the result is released, and how often a sample may be queried. They do not prove that the encrypted SNPs came from the registered sample, that the model is clinically valid, or that repeated queries reveal no information about private weights.

We consider a computationally bounded adversary with full network access to the blockchain and coprocessor (Fig. 5). The adversary may observe all on-chain transactions and storage slots; operate a validator node and read all encrypted handles; submit arbitrary transactions; and query classification outputs repeatedly with chosen SNP inputs. A requester who is allowed to use private weights may submit values that do not come from the registered sample. The adversary may not break the TFHE hardness assumption or bypass the contract rules about who may use data and receive results.

### Source of the encrypted SNPs

The registry checks whether a person may request a calculation, but it cannot check whether the encrypted SNP values came from the registered biological sample. The contracts calculate a result from the values they receive, including values chosen by a requester. Restricting an attack to correlated SNP patterns is therefore an analysis choice, not a restriction enforced by the system.

This study assumes that the patient, laboratory, or data holder prepares the genotype data correctly before encryption, following Section Genotype preprocessing, QC, and model alignment. The stored sample record describes the genome build, variant order, preparation rules, and a cryptographic fingerprint of that record. It helps document how the input was prepared, but it does not prove that the encrypted values came from the stated biological sample.

### Core privacy invariants

Under the assumptions in Table 2, we formalize the protocol’s privacy properties as five invariants:

**Table 2:** Parts of the system on which privacy, correctness, availability, and the sample or model record depend. A mark shows what could be affected if that part fails.

Component	Privacy	Correctness	Availability	Record	Responsibility
Genotype provider/preprocessor		X		X	prepares the claimed sample and applies build, order, QC, and orientation rules
Model provider		X		X	publishes the intended weights, metadata, thresholds, and scientifically valid model
Smart contracts	X	X	X	X	apply the stated calculation and the rules about who may use data or receive results
Blockchain consensus		X	X	X	orders and finalizes the publicly auditable contract record
fhEVM coprocessor	X	X	X		performs TFHE operations correctly without exposing plaintext
Relayer	X		X		carries requests without revealing a released result to someone else
Decryption services	X	X	X		enforce raw-score decryption permissions and serve public category decryption

Invariant 1. (Ciphertext opacity). Encrypted handles are 32-byte opaque identifiers; raw ciphertexts reside exclusively at the off-chain coprocessor. No on-chain party stores or observes plaintexts at any point during computation.

Invariant 2. (ACL-gated decryption). Every encrypted output is associated with an ACL record. Decryption requires an explicit ACL grant that can only be issued by the executing smart contract upon satisfying the protocol’s state-machine preconditions.

Invariant 3. (No raw score release). The final encoded PRS *e* (Equation 6) is never made publicly decryptable. When oracle-required mode is disabled, the requester may obtain ACL-gated decryption access to the raw score through finalize(). When oracle-required mode is enabled, that requester path is disabled: the raw score handle is routed only to the approved oracle, which emits a publicly decryptable randomized category.

Invariant 4. (Oracle-required mode). A model owner may set an oracle-required flag before finalization, causing the compute engine to revert on any attempt to release the raw score handle directly to the requester. This prevents requesters from bypassing the randomization by decrypting raw scores. When oracle-required mode is active, only the registered approved oracle address may receive the encrypted raw-score handle.

Invariant 5. (Single-finalize guarantee). Each compute job carries a finalized flag. Any finalization path sets this flag and reverts on subsequent calls, preventing a requester from obtaining multiple score handles for a single rate-limit slot.

For models that do not require the oracle, the legacy readPartial function can separately expose an intermediate accumulator to the requester; this path is outside the finalization invariants and was not used in the evaluation. These invariants do not establish where the submitted SNPs came from, whether the model is clinically valid, or whether the external fhEVM services behave correctly.

### Randomized risk category

Before assigning Low, Medium, or High risk, the Result contract adds a random nonnegative integer smaller than *B* to the encoded score:


(8) \begin{eqnarray*}& \begin{aligned} e_{\textrm{randomized}} &= e + \nu,\\ \nu &\sim \operatorname{DiscreteUniform}\{0,1,\ldots,B-1\}. \end{aligned}\end{eqnarray*}

The fhEVM supplies *ν*; the requester does not choose it. The oracle deployer fixes *B*, a power-of-two immutable constant, in the oracle constructor. Before model finalization, the model provider selects an oracle and fixes the two category thresholds; requesters cannot change those values between queries. This makes it harder to narrow down an encrypted score by repeatedly changing a comparison threshold.

All comparisons against classification thresholds operate on the randomized encrypted score using encrypted boolean operations, avoiding any branching on hidden values:


(9) \begin{eqnarray*} \hat{L} &= \mathbb{1}[e_{\textrm{randomized}} < \tau_{L}], \notag\\ \hat{H} &= \mathbb{1}[e_{\textrm{randomized}} \geq \tau_{H}], \notag\\ \textrm{cat} &= \textrm{select}\bigl(\hat{L},\;L,\;\textrm{select}(\neg\hat{L}\wedge\neg\hat{H},\;M,\;H)\bigr).\end{eqnarray*}

The encrypted select primitive replaces conditional branching, preventing side-channel leakage of the comparison result. The minimum threshold-gap requirement $\tau _{H} - \tau _{L} \geq B$ ensures that the randomized score cannot map deterministically to a single category regardless of *e*.

**Interpretation and limits.** This method does not provide an $(\varepsilon ,\delta )$-differential privacy guarantee. It uses one-sided noise, does not relate *B* to a formal measure of how much one input can change the score, and does not account for information from repeated queries. A formal privacy method would need to address each of these points.

**Bias correction.** Because the random value is always nonnegative, its exact mean is *(B-1)/2*. The contract provides the integer threshold correction *B/2*; for *B=128*, this is 64, while the exact mean is 63.5. Scores close to a threshold remain uncertain. In the 100-SNP study, 48 individuals outside this uncertainty range kept the same category. The other two were each 64 units below a threshold, so their category could change. The *B=128* category-agreement and repeated-query results were obtained only in the local simulation. The deployment script uses $B=2^{20}$ for its general oracle deployment, and we did not evaluate a randomized category release on Sepolia.

### Analysis of repeated queries

Without query limits, an adversary could attempt a model-extraction attack by submitting many classification queries with crafted SNP inputs and observing the resulting categories. The compute engine therefore enforces per-model, per-wallet, and per-sample job quotas over a window of *W* blocks, admitting at most *R* queries per window. Block-based windows, rather than timestamps, prevent miner manipulation of window boundaries.

We examined whether a requester allowed to use a model with 20 private weights could infer the weights by submitting chosen encrypted SNP values and observing the released results. The analysis included thresholds chosen by the requester, with each query chosen after seeing earlier results or all queries chosen in advance; thresholds fixed by the model provider; several wallets and samples; and correlated SNP patterns. These calculations were performed in the local simulation and are summarized in Table 3. To make the analysis repeatable, we used a fixed sequence of random integers from 0 through 127. A different random sequence may give different exact recovery counts. “Within *B*” means that an estimated integer weight differs from the true integer weight by less than 128 units; it does not mean exact recovery. Pearson *r* measures whether the estimated weights preserve the true relative pattern, and sign accuracy is the fraction whose positive or negative direction is correct. The 320-query rows use a common comparison budget rather than a protocol security threshold.

**Table 3:** Results of the repeated-query analysis in the local simulation. “Within *B*” is the number of weights estimated to within the randomization range.

Information released and query choice	Queries	Pearson *r*	Sign acc.	Within *B*
Raw score available	20	1.0000	100%	20/20
Requester changes threshold after each result	200	0.9999	100%	19/20
Requester changes threshold after each result, first 20/20	260	1.0000	100%	20/20
Requester-selected thresholds, queries chosen in advance	320	0.6689	65%	0/20
Thresholds fixed by model provider	320	0.9388	70%	0/20
Fixed thresholds with correlated SNP blocks	320	0.0223	65%	0/20

In this analysis (Table 3), changing the threshold after each result recovered 19 of 20 weights within *B* after 200 queries and first recovered all 20 after 260 queries. When all queries were chosen in advance, none was recovered within *B* after 320 queries (*r=0.6689*; 65% of signs correct). With thresholds fixed by the model provider, none of the 20 estimates was within *B* after 320 queries. However, Pearson *r=0.9388* and 70% sign accuracy show that the results still revealed some information about the relative weights. The fixed thresholds therefore made precise recovery harder but did not completely hide the private weights.

Using several wallets did not increase the number of queries allowed for the same sample, although different registered samples had separate limits. For private weights, the model provider decides who may use them. When inputs were restricted to correlated SNP blocks, the correlation between estimated and true weights fell to *0.0223*. This is not a reliable safeguard because requesters can still submit other encrypted SNP values. Applying the studied limit of three calculations per 1000 blocks to 260 total queries gives calculated times of 288.9 h with 12-s blocks or 48.1 h with 2-s blocks for one registered sample. These are calculated examples, not measured network times. The chosen *B=128* was 1.34% of the largest integer weight; other models may require a different value. The model owner can update or disable the rate limits after publication, so their protection depends on the maintained configuration.

## Empirical evaluation

### Where calculations were evaluated

We report results from Sepolia and from a local contract simulation. Sepolia provides observed fhEVM transaction results. The local simulation evaluates contract behavior, transaction counts, and gas used in a local contract environment; it does not measure real encrypted-computation time, Sepolia capacity, or production fees.

The archived local evidence records Node.js 22.23.1 on macOS. The contract build uses Solidity 0.8.24, Hardhat 2.22.2, @fhevm/hardhat-plugin 0.4.2, and @fhevm/solidity 0.11.1; the resolved dependency versions are retained in package-lock.json. The Solidity optimizer used 200 runs with viaIR enabled and the Cancun EVM target, as recorded in hardhat.config.ts.

We evaluated public-weight local calculations against the four fixture sizes (100, 500, 1000, and 5000 SNPs) from the HEPRS reference dataset [7], which contains real GWAS beta weights for a schizophrenia model at each size, and one private-weight local calculation with 100 SNPs. Each public data set also includes one leading constant position. The largest successful calculation chunk in the local simulation contained 21 SNPs for both public and private weights; all experiments use upload chunk size $r_{u} = 32$ and compute chunk size $r_{c} = 20$. The corresponding Sepolia limit was not measured.

On Sepolia (chain ID 11155111), a public-weight 100-SNP calculation completed in 25 transactions after four contract deployments. It used 20.710271 million gas, took 269 320 ms from submission to result and 8081 ms to decrypt, and returned an encoded score of 758 685. This score exactly matched the independent calculation. The private-weight 100-SNP calculation was evaluated locally but was not run on Sepolia.

### Agreement with an independent calculation

We compared all 200 public-weight results with an independent calculation of Equation 1: 50 individuals at each of the four model sizes. The mean, root-mean-square, and maximum absolute errors were all zero; all 200 scores matched exactly; and Pearson *r=1*. This supports the calculation for the studied inputs.

The exact agreement is not a general precision result. The four data sets contain 6600 variant weights plus four leading constant weights. All 6604 values have at most six decimal places, and the selected scale represented them without rounding. Other weight sets may show small rounding differences.

For the randomized risk category at 100 SNPs, all 48 individuals outside the threshold uncertainty range kept the same category. Two individuals were inside the range and are reported separately; one changed category in this calculation.

### Variant scale

Table 6 reports the number of transactions used for one public model and one sample. The 100-SNP Sepolia result used the Classic method (stored inputs). The local results from 100 to 5000 variants used the Streaming method; 5000 variants was the largest bioETH-PRS model evaluated.

**Table 4:** Sepolia and local results for the same public-weight 100-SNP calculation using the Classic method (stored inputs), upload groups of 32, calculation groups of 10, and 25 transactions. Sepolia used 10.42% more gas than this one local simulation; this percentage is not a general conversion between local and network results.

Setting	Tx	Gas	Submit–result	Decrypt	Encoded score
Sepolia	25	20.710271 M	269 320 ms	8081 ms	758 685
Local simulation	25	18.755864 M	147 ms	125 ms	758 685

**Table 5:** Independent Equation 1 calculation versus decoded public-weight bioETH-PRS in the local simulation. The study weights were represented without rounding.

SNPs	Scale *s*	*n*	MAE/RMSE/max	Exact
100	$3\times 10^{6}$	50	0/0/0	50/50
500	$3\times 10^{6}$	50	0/0/0	50/50
1000	$1\times 10^{6}$	50	0/0/0	50/50
5000	$1\times 10^{6}$	50	0/0/0	50/50

**Table 6:** Transactions for the public-weight calculation on Sepolia and in the local simulation.

Setting	Variants	Method	Tx
Sepolia	100	Classic	25
Local simulation	100	Streaming	15
Local simulation	500	Streaming	47
Local simulation	1000	Streaming	88
Local simulation	5000	Streaming	413

### Gas consumption and scaling

**Figure 6:**
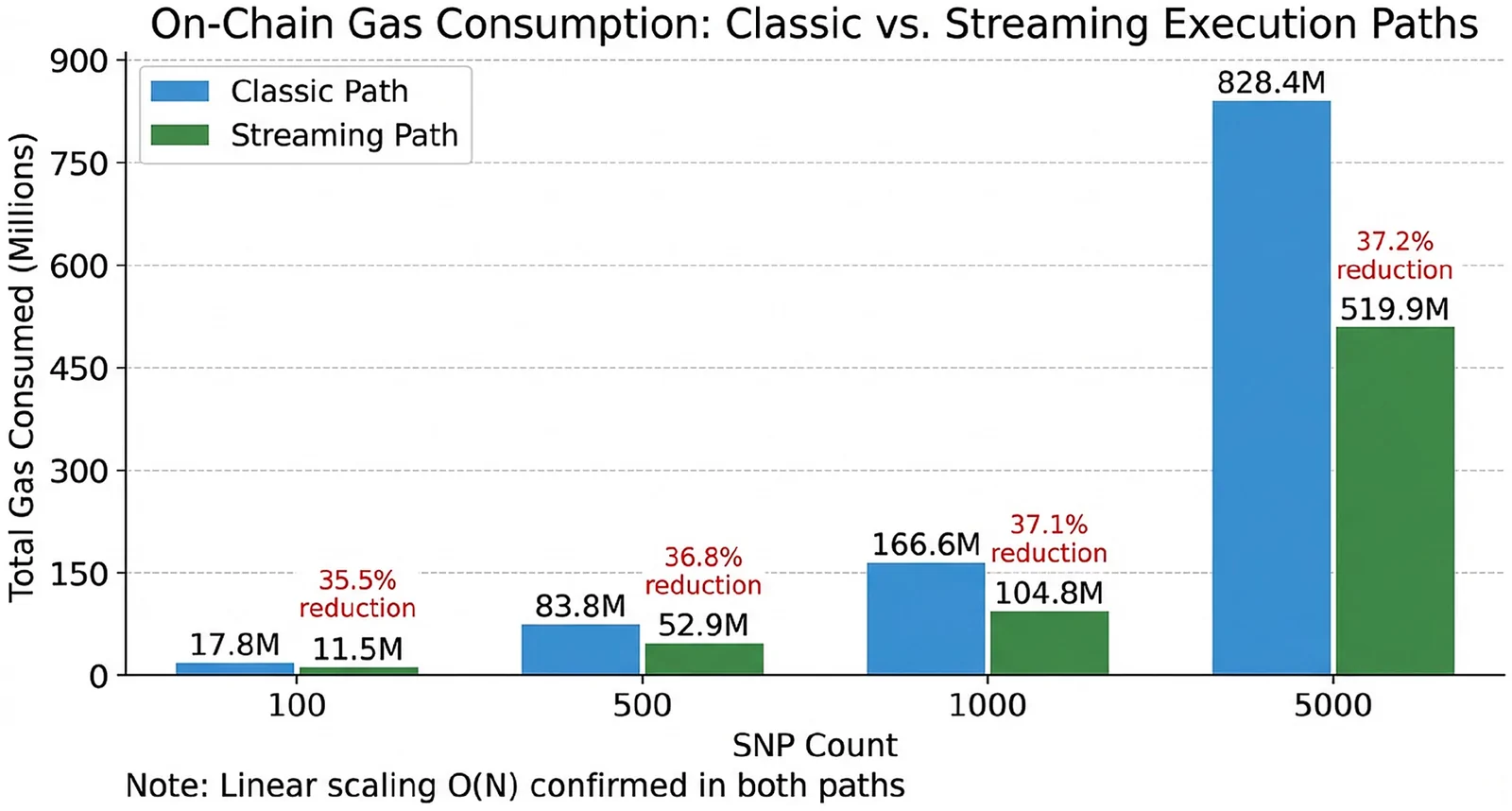
Original gas-scaling visualization for the two local calculation methods. The graphic retains the original plotted values; Table 7 reports the final post-review measurements and is the numerical source for the revised comparison.

Figure 6 and Table 7 report gas used by the two calculation methods for four public-weight calculations in the local simulation. Gas grows approximately linearly over the measured 100–5000-variant range; this is not a claim about scaling on Sepolia. Across this range, the Streaming method used 35.4%–37.2% less gas because it did not store each encrypted SNP reference permanently.

**Table 7:** Gas used in one local calculation, rounded to 0.001 million. The classic and streaming totals include model publication and result release but exclude sample registration. Both methods used calculation chunks of 20 SNPs.

SNPs	Classic	Streaming	Reduction
100	17.863 M	11.538 M	35.4%
500	83.985 M	53.063 M	36.8%
1000	166.875 M	105.013 M	37.1%
5000	829.422 M	520.487 M	37.2%

### Per-SNP cost decomposition

Table 8 decomposes per-SNP gas in the local simulation. In the present implementation, input-proof verification, the FHE multiply, and the FHE accumulate account for *∼*95 000–104 000 gas per SNP before persistent storage is considered—operations set by the fhEVM protocol rather than by contract design choices. Changes to the fhEVM services or input preparation could change these values. These component values are engineering estimates derived from the operation pattern and total-gas differences; they were not measured as independent transaction components.

**Table 8:** Engineering estimate of gas used for each SNP in the local simulation.

Operation	Classic	Streaming
Input proof verification	*∼* 50 000	*∼* 50 000
FHE multiply	*∼* 27 000	*∼* 27 000
FHE accumulate (partial sum)	*∼* 27 000	*∼* 27 000
Handle SSTORE (classic only)	*∼* 25 000	—
ACL SSTORE (classic only)	*∼* 25 000	—
SLOAD, misc. overhead	*∼* 12 000	—
**Total/SNP**	*≈* **166 000**	*≈* **104 000**

### Latency

Local-simulation times for each public-weight calculation were 157, 780, 1,672, and 8,819 ms for 100, 500, 1000, and 5000 variants, respectively. Each data set also includes one leading constant position. The measured range was 1.554–1.763 ms per position. The time per position increased by about 13% between the smallest and largest local calculations. These times combine the local arithmetic and transaction overhead; they measure neither real TFHE evaluation nor network latency. On a real network, latency is dominated by per-block confirmation time, as each chunk corresponds to one on-chain transaction; the transaction count scales as $\lceil N / r_{c} \rceil + 2$ for the streaming path. The separate Sepolia 100-SNP times appear in Table 4 and are not compared as a speed ratio.

### Transactions, gas, and fee examples

Table 9 reports the observed transaction use. The Sepolia deployment used four transactions and 5.892559 million gas, costing 0.0062781714 Sepolia test ETH; the public-weight calculation used 25 transactions and 20.710271 million gas, costing 0.0252747648 test ETH. These are test-network expenditures, not production prices. In the local simulation, a public-weight calculation with 100 SNPs using the Streaming method used 15 transactions and 11.690 million gas, whereas the private-weight calculation with 100 SNPs used 17 transactions and 23.508 million gas (2.01*×*). The private-weight calculation is the one relevant to attempts to recover the weights. Decryption happens outside Ethereum and requires no additional Ethereum transaction.

**Table 9:** Measured transaction use. The local streaming totals include sample registration and come from a separate calculation from Table 7; encrypted inputs also cause small gas variation between otherwise identical calculations.

Setting	Quantity	Tx	Gas
Sepolia	four-contract deployment	4	5.892559 M
Sepolia	public-weight 100-SNP, Classic method	25	20.710271 M
Local simulation	public-weight 100-SNP, Streaming method	15	11.690 M
Local simulation	private-weight 100-SNP, Streaming method	17	23.508 M
Local simulation	one-time category settings	1	0.077 M

Recording a digital fingerprint for the model file and another for its preparation information added 40 568 local gas per model. This added no encrypted arithmetic and did not change the largest successful local calculation group. The randomized category settings require one additional model transaction (77 314 local gas). Releasing a raw score used 169 898 local gas, whereas releasing a randomized category used 432 230 local gas. These are alternative ways to release one finalized result and are not added together.

For illustration only, we multiplied three local gas measurements by hypothetical gas prices: deployment (5892 613 gas), the public streaming calculation (11 690 021 gas), and the private streaming calculation (23 507 880 gas). These are calculated estimates, not observed costs. At 1 gwei, the respective values are 0.005892613, 0.011690021, and 0.02350788 ETH; at 30 gwei, they are 0.17677839, 0.35070063, and 0.7052364 ETH. We make no USD conversion or conclusion about affordability or clinical or commercial use.

### Calculation checks and responsibilities

The independent comparison summarized above shows agreement with Equation 1 for the studied inputs; it is not a guarantee for every model or deployment. Table 10 explains who is responsible for each part of the result.

**Table 10:** Responsibilities and remaining limits.

Actor/component	Responsibility in this study	Remaining limit
Data preparer	check genome build, variant order, dosage validity, missing values, and effect-allele alignment	the contracts do not prove that the encrypted SNPs came from the stated biological sample
Model provider	supply the weights, model details, thresholds, and scientific justification	calibration or performance in populations that were not studied
Smart contracts	apply the stated integer calculation and rules about who may use data or receive results	the contracts cannot verify input claims or external services
fhEVM services	perform the encrypted arithmetic and apply the configured release permissions	blockchain consensus does not independently verify the encrypted arithmetic
Independent calculation	compare Equation 1 with 200 studied inputs	agreement is not a proof for other models, inputs, or deployments
Requester	inspect the published model details and blockchain transactions	clinical interpretation beyond the published model

The contracts check who may receive a finalized result, allow only one finalization per job, keep the thresholds fixed by the model provider, require the minimum threshold gap, apply query limits, and stop a private-weight calculation when the model provider no longer allows it. The legacy readPartial path described above is separate from finalization and is blocked only when oracle release is required. These protocol invariants—ACL enforcement, state-machine integrity, the single-finalize guarantee, the minimum threshold gap, oracle-required mode, approved-oracle enforcement, rate-limiting window behavior, and ACL revocation handling—were validated across 188 automated test cases. The contracts do not establish the source of the biological sample, clinical validity, calibration, or performance in other populations.

## Access control and compute flows

### Encrypted handle lifecycle

Every encrypted value in the fhEVM has an associated ACL record maintained in a dedicated on-chain contract. The ACL maps handles to authorized addresses and distinguishes four grant types with different persistence semantics:

- **Persistent contract grant**: Written to the ACL contract’s persistent storage via SSTORE. Allows the owning contract to use the handle in future transactions. This is the primary source of gas cost in the classic upload path (*≈*25 000 gas per SNP handle).
- **Persistent user grant**: Written to the ACL contract via SSTORE. Allows an end-user to decrypt the handle via the decryption services. Applied at finalization to authorize the requester for their encoded score.
- **Transient grant**: Valid only for the current transaction. The streaming path creates no persistent grant for an intermediate SNP handle, avoiding the SSTORE cost entirely.
- **Public grant**: Enables anyone to decrypt. Applied only to the randomized risk category; never applied to score values.

The ACL design is the root of the gas differential between the classic and streaming paths. In the classic path, each of the *N* SNP handles requires a persistent contract grant (SSTORE in the ACL) and a handle write to contract storage (second SSTORE). In the streaming path, neither persistent write is performed. A finalized raw score is authorized only for the requester; if the model requires a risk category, the randomized Low, Medium, or High result is made publicly decryptable. For raw-score models, readPartial can also authorize the requester to inspect an intermediate accumulator before finalization.

### State machine and mutual exclusion

The PRS Compute Engine enforces a strict job state machine to prevent incorrect ordering of operations:


\begin{align*}& \mathtt{PENDING} \!\to\! \mathtt{UPLOADING} \!\to\! \mathtt{READY} \!\to\! \mathtt{COMPUTING} \!\to\! \mathtt{DONE}. \end{align*}

The classic and streaming paths are mutually exclusive per job. The guard condition uses upload counts: if any SNP handles have been written to persistent storage, the classic path is in use and streaming calls are rejected; if computation has advanced without persistent uploads, the streaming path is in use. This prevents hybrid calls that could compromise the handle-lifecycle invariants.

### Private model access control

When a GWAS model is published with private (encrypted) weights, access control is layered: the model owner must first authorize the compute engine as a reader; individual requesters must also be added to the private model reader list; and each compute chunk re-checks reader authorization, so revocation of a requester mid-job causes the next compute chunk to fail, effectively terminating the job. This is in contrast to the registry ACL, which is checked only at job creation. The asymmetric behavior between registry and model ACL is documented and empirically verified.

## Discussion

We evaluated bioETH-PRS with additive PRS models containing up to 5000 variants. Publicly auditable smart contracts reduce reliance on a single designated evaluator, but the system still depends on the blockchain and on fhEVM services that perform encrypted calculations, submit transactions, and apply the configured release permissions. A public-weight 100-SNP calculation completed on Sepolia. Public-weight calculations with 100–5000 variants and one private-weight 100-SNP calculation were evaluated in the local simulation. These results do not establish genome-wide or clinical use.

### HEPRS and bioETH-PRS: complementary systems

bioETH-PRS and HEPRS [7] address different questions and are best understood as complementary rather than competing systems.

HEPRS reports an encrypted PRS calculation for a substantially larger model and measures CKKS performance under a three-party non-collusion assumption. CKKS represents approximate signed decimal values without the integer conversion used here. HEPRS therefore provides the relevant results for large variant counts and measured FHE performance.

bioETH-PRS instead studies whether public smart contracts can coordinate the calculation and control result release without a designated evaluator. Its model settings and transaction history are public, while the system still relies on the fhEVM services. It also requires many blockchain transactions. The two systems answer different questions, and these results do not show that either is broadly superior.

### Limitations and open problems

**SNP count ceiling.** The largest successful calculation chunk in the local simulation contained 21 SNPs for both public and private weights; the Sepolia limit remains unknown. A public-weight 5000-variant calculation required 413 local transactions. We did not evaluate larger bioETH-PRS models or production feasibility.

**SNP provenance.** As specified in Section Security assumptions and limits, being allowed to request a calculation does not prove that the encrypted SNP values came from the registered sample.

**URI observability.** Sample data URIs are visible on the blockchain. Any network participant can read them, even if the application limits who may use the sample. Storing only a cryptographic fingerprint of the URI would reduce this exposure.

**One-sided randomization and bias.** Adding an integer chosen uniformly from *0* through *B-1* increases the score by *(B-1)/2* on average and creates an uncertainty range near each threshold. This randomized category result is not differential privacy because the random value is one-sided, is not formally calibrated to score sensitivity, and has no formal composition accounting across repeated queries.

**Intermediate raw accumulator.** When oracle release is not required, the legacy readPartial function permits the requester to inspect an intermediate encrypted accumulator. It is blocked for oracle-required models and was not used in the reported evaluation, but it is an additional raw-output channel and must be included in any deployment-specific probing analysis.

**Production cost.** We report gas used by the contracts, Sepolia test-ETH expenditure, and calculated fee examples using hypothetical gas prices. Production fee schedules, USD cost, memory use, and operational throughput were not measured, so whether the system is affordable or practical for clinical or commercial use remains unknown.

### Future directions

Future work should reduce the number of blockchain transactions and evaluate the private-weight 100-SNP calculation on Sepolia. Establishing the source of encrypted SNPs will require a signed laboratory record or a privacy-preserving proof that links the encrypted values to the registered sample. A formal privacy guarantee will require a precise definition of which data sets are compared, how much one input can change the score, how noise is chosen, and what repeated queries reveal.

Further planned extensions include (i) LD-aware weighting in the encrypted domain, incorporating LDpred2-style shrinkage [10]; (ii) multiparty key generation enabling federated cohort analyses without a single decryption authority; (iii) model versioning and deprecation mechanisms; (iv) SNP handle reuse across multiple models from the same registered sample; and (v) token-based fee mechanisms to economically incentivize model quality. The architecture’s modularity—four independently deployable contracts—is designed to accommodate these extensions without disrupting existing deployments.

## Related work

Privacy-preserving genomic computation has been pursued through multiple cryptographic paradigms. Kim and Lauter [14] applied homomorphic encryption schemes to compute GWAS statistics for up to 5000 sequences, but did not extend to PRS computation. Blatt *et al*. [15] built a homomorphic-encryption pipeline for large-scale GWAS and reported PRS values derived from the encrypted association tests. McLaren *et al*. [16] demonstrated privacy-preserving genomic testing in an HIV clinical-care model using homomorphic encryption, but did not address the full PRS pipeline. Raisaro *et al*. [17] introduced MedCo for secure, privacy-preserving exploration of distributed clinical and genomic data.

Knight *et al*. [7] (HEPRS) is the closest prior work, providing a complete FHE pipeline for PRS with real clinical data. HEPRS achieves *r> 0.999* correlation with plaintext scores on a 110 000-SNP schizophrenia model and demonstrates practical feasibility on commodity hardware. bioETH-PRS uses the HEPRS study fixtures and the same additive PRS definition, studying a different trade-off in which public contracts coordinate the calculation while infrastructure trust remains.

On the blockchain side, prior work on privacy-preserving smart contracts has focused on anonymous payments and related protocol work [18, 19] and zero-knowledge proof-based computation [20]. Zero-knowledge proofs can verify a calculation while keeping designated witness data private, whereas FHE allows calculations directly on encrypted data. FHE on the blockchain, typified by the fhEVM framework [13], combines confidential operations with public smart-contract records while depending on external encrypted-computation services. bioETH-PRS applies this framework to PRS calculation.

## Conclusion

We presented bioETH-PRS, a protocol for confidential polygenic risk scoring using TFHE-based FHE on a programmable blockchain. Publicly auditable smart contracts coordinate the calculation without a designated evaluator. This reduces reliance on one evaluator but does not remove trust: the result still depends on genotype preparation, model validity, the contracts, blockchain, and fhEVM services that perform encrypted calculations and apply the configured release permissions. Raw scores are authorized only for the requester, whereas randomized categories are publicly decryptable.

One public-weight 100-SNP calculation completed on Sepolia and matched the independent calculation exactly. Public-weight calculations with 100–5000 variants and one private-weight 100-SNP calculation were evaluated only in the local simulation. All 200 public-weight local scores matched Equation 1 exactly because the study weights were represented without rounding. The Streaming method used 35.4%–37.2% less local gas. In the repeated-query analysis, thresholds fixed by the model provider and query limits made precise weight recovery harder, but some information about relative weights remained. The randomized category result does not provide differential privacy.

The results apply to additive PRS models containing up to 5000 variants. This study does not establish genome-wide or clinical use, production affordability, private-weight calculation on Sepolia, or the largest calculation step that Sepolia can support. HEPRS demonstrates encrypted calculation at a larger scale; bioETH-PRS studies how smart contracts can coordinate a smaller calculation and control result release.

**Key Points**

- Smart contracts reduce reliance on a single designated evaluator, but the system still depends on the blockchain and fhEVM services.
- A public-weight 100-SNP calculation completed on Sepolia. In the local simulation, we evaluated public-weight calculations with 100–5000 variants and one private-weight 100-SNP calculation.
- All 200 locally decoded public-weight scores matched Equation 1 exactly because the study weights were represented without rounding.
- The Streaming method used 35.4%–37.2% less gas than the Classic method (stored inputs) in the local simulation.
- Randomized risk categories and query limits make exact recovery of private weights harder, but they do not provide differential privacy or completely hide the weights.

## Data Availability

No new datasets were generated. We analyzed the public HEPRS fixtures retained under test/fixtures/heprs/; the derived validation and measurement records are retained under evidence/ in the study repository. The code, fixtures, validation scripts, and result records used in this study are available at https://github.com/Georgakopoulos-Soares-lab/bioETH-PRS.
